# Pressure Anesthesia

**Published:** 1905-04-15

**Authors:** J. V. Conzett


### PRESSURE ANESTHESIA.

BY J. V. CONZETT, D.D.S.

Some one has said that pressure anesthesia is the greatest advance that dentistry has made in the last twenty years. Whether that be so or not, it certainly is a great blessing to the patient and a boon to the operator. Inasmuch as Dr. Richardson has given you a paper in which he very ably describes the technique of the operation, it will not be necessary for me to attempt to do so. I shall content myself with a few observations upon its use, abuse, and limitations. It is the legitimate child of cataphoresis, and if that much-abused method did nothing else during its short career than beget vapo-cocain, which begat pressure anesthesia, it has not lived in vain.

It might be interesting and, I believe, highly instructive, had we the time and talent, to study the action of pressure anesthesia; how it acts upon the contents of the destinal tubules, producing in some instances a perfect anesthesia upon the erstwhile sensitive dentin, and then again disappointing us be producing no effect whatever. What is its action? What are the contents of the tubules? Nerve filaments or protoplasm? How do they propagate the sensation? I do not know, and not knowing, do not know how to obtain perfect results. In some instances in which I have used pressure anesthesia for sensitive dentin I have been delighted with its success, and then again could gain absolutely no results whatever. Is there a difference in the size of the tubules that in one case allows the medicament to penetrate and in the other refuses it passage? Or is there a certain elusive something in the chemical constitution of the contents of the tubules that refuses in one case to be anesthetized and in another, being absent, the pulp promptly goes to sleep? I only know that sometimes pressure anesthesia is successful in desensitizing dentin and sometimes it is not. But when it comes to pulp extirpation—ah! that is another story. Here is where it smiles upon us and assures us of immediate and perfect success. I use this method in fully ninety-five percent of my cases, in all teeth and in nearly all conditions of decay of said teeth. So much for its use.

Is it abused? Yes, like all good things, it is sometimes used indiscriminately and unintelligently and the results are bad. It is used in some instances without disinfecting the cavity or making any attempt at such a desirable result, and then the resulting soreness of the peridental membrane and subsequent abscess is laid at the door of the method instead of the operator. Never use pressure anesthesia until you have as thoroughly as possible disinfected your cavity. Remove all the decay you possibly can, and then wash the cavity with alcohol, dry carefully, and if you have no exposure use formalin, flooding the cavity therewith. If you have an exposure or an inflamed pulp upon which formalin would have too irritating an effect, flood the cavity with a ten percent solution of chinisol. In either case allow the drug to remain in the cavity for two or three minutes, then gently evaporate the drug with warm air, then proceed with the pressure. Another point to remember is not to force the cocain solution through the apical foramen. I believe that that is a frequent cause of soreness. It is not necessary to anesthetize the apical space beyond the tooth. Many advise the immediate filling of the root-canal and give a reason for the faith that is in them. Personally, I do not do that. I believe that when we anesthetize a pulp and remove it that we cause such a shock to the contiguous tissues that they need time for recovery. Moreover, there is in a large percentage of the cases of persistent hemorrhage, which it is inadvisable to suddenly check, and if not checked and filled you have blood in your canal to give future trouble. Even in those cases in which there is no hemorrhage there is a seeping in of a greater or less amount of serum into the pulp-canal, the filling of which in such cases is not good practice; and then last, but perhaps the most important reason, is that we are not always sure that every trace of the pulp has been removed, and if a filling is placed in the canal impinging upon a live shred of the pulp we will have a beautiful case of trifacial neuralgia.

I have found a little sensation at the apex on the second sitting, and upon investigation and a little cocain judiciously used have removed therefrom a small piece of the pulp that had not been removed. In such cases I am always glad that I did not fill the canal at the first sitting. But perhaps I am the only one that ever had any difficulty of that kind.

After removing the pulp, saturate a piece of cotton with oil of cloves, and with a hot-air syringe held close to the cotton evaporate the oil of cloves, some of which will penetrate the tubules and apical foramen and will give a sense of comfort to the patient and largely obviate the chances of after-soreness.

Has it any limitation?

I will admit that that depends a great deal upon the operator. I believe that with some men there would be practically no limitations. Again with other the limitations would exceed the indications. There are some cases, however, in which I never use it. I never use it on a pulp that is in a septic condition. We sometimes see pulps that are in a necrotic condition while that portion in the canals is alive. I do not use pressure in those cases, fearing to force the septic material through the apical foramen and setting up an alveolar abscess. I believe in such cases it is better to use arsenic. Some say that it is contra-indicated in highly-inflamed pulps, but I have had so much success there that I connot concur in that statement. An inflamed pulp can be successfully removed by pressure anesthesia if you have the time and the patience to give to it. If not, don't try it.

Then there is the constitutional limitation, if I may so call it: those cases in which there is an idiosyncrasy which contra-indicates the use of cocain in any degree. I confess to a hestitancy to use cocain for a red-headed person, especially if it is a child. Why cocain and atropin as well should not be as well borne by those of a light complexion as those of a dark complexion is a mystery to me, but it seems to be a fact. There are some cavities that do not lend themselves well to pressure anesthesia by reason of location, but in most cases this difficulty can be obviated by building up with gutta-percha or cement.

In conclusion I most heartily endorse the method, and earnestly advise any that have not used it or do not use it, to any great extent, to investigate the method, carefully progressing until they master the details thereof, and thereafter it will be a source of joy to them forever.

